# Expression and Diagnostic Value of miR-497 and miR-1246 in Hepatocellular Carcinoma

**DOI:** 10.3389/fgene.2021.666306

**Published:** 2021-06-07

**Authors:** Shuying Chen, Zile Fu, Shuzhan Wen, Xiaoyi Yang, Chengxuan Yu, Wenhan Zhou, Yong Lin, Yuan Lv

**Affiliations:** ^1^Department of Laboratory Medicine, Huashan Hospital, Fudan University, Shanghai, China; ^2^Shanghai Medical College, Fudan University, Shanghai, China; ^3^School of Medicine, Shanghai Jiao Tong University, Shanghai, China

**Keywords:** hepatocellular carcinoma, microRNA-497, microRNA-1246, biomarker, diagnostic value, target genes

## Abstract

**Objective:**

Serum microRNAs (miRNAs) may serve as biomarkers in various cancers. Our study aims to explore the roles of miR-497 and miR-1246 in hepatocellular carcinoma (HCC).

**Methods:**

The expression levels of miR-497 and miR-1246 were measured by RT-PCR. A correlation analysis was conducted between the expression levels of miR-497 and miR-1246 and clinicopathological characteristics of patients. The receiver operating characteristic (ROC) curve was applied to evaluate the diagnostic efficacy in HCC. In addition, bioinformatics tools were also utilized to predict the potential targets of miR-497 and miR-1246.

**Results:**

The expression level of miR-497 in HCC was significantly down-regulated compared with the control group while the miR-1246 revealed a significantly higher expression level in HCC. There was a significant correlation demonstrated between the expression levels of miR-497 and miR-1246 in preoperative serum of HCC and the differentiation degree, Tumor Node Metastasis (TNM) classification, and metastasis. The expression levels of serum miR-497 and miR-1246 were significantly associated with the diagnosis, prognosis, and overall survival rate of patients with HCC. Moreover, the potential target genes of miR-497 in HCC include ARL2, UBE2Q1, PHF19, APLN, CHEK1, CASK, SUCO, CCNE1, and KIF23. The low expression of these nine genes is associated with a better prognosis of HCC patients. AUTS2 is a novel target gene of miR-1246, and its low expression is significantly related to the low overall survival rate of HCC patients.

**Conclusions:**

miR-497 and miR-1246 are possibly involved in the progression of HCC by regulating target genes, respectively, and could serve as biomarkers in HCC.

## Introduction

Hepatocellular carcinoma (HCC) is one of the most prevalent cancers in the alimentary system. It ranks sixth in terms of morbidity and fourth on the list of cancer-related deaths worldwide. Due to the remained difficulties in identifying early-stage cases and the high possibility of tumor recurrence and metastasis after resection operation, HCC still poses a huge threat to human health and survival (Villanueva, 2019). Hence, profound study into the mechanism of tumorigenesis and the discovery of biomarkers with high sensitivity and specificity aiming HCC for the early diagnoses and prognosis monitoring are both essential.

MicroRNAs (miRNAs) are short, single-stranded non-coding RNAs of 18∼22 nucleotides. They down-regulate the expression levels of target genes post-transcriptionally through binding to the 3′untranslated region (3′UTR) of their mRNAs, by which way they modulate various cellular events including apoptosis, cell cycle, proliferation, and invasion and play key roles in tumorigenesis and malignant development (Gebert and MacRae, 2019; Treiber et al., 2019). Recent researches have discovered that altered expression levels of miRNAs in HCC are bound up with biological behaviors and clinicopathological characteristics, making miRNAs pivotal factors in tumorigenesis of HCC (Borel et al., 2012). For example, miR-30a-5p can inhibit glycolysis and increase sensitivity to sorafenib through targeting CLCF1 in HCC, indicating modulating the expression level of miR-30a-5p has a promising therapeutic effect on HCC patients (Zhang et al., 2020). Meanwhile, miR-145-5p can promote tumor progression by inhibiting SPATS2 (Dong et al., 2020). In view of this research progress, miRNAs attract increasing research interest as potential targets in HCC diagnosis and treatment.

Recently, we found that miR-497 and miR-1246 differed in the expression of hepatocellular carcinoma by a large number of literature searches. Several studies focused on the effects of miR-497 in tumor growth (Chae et al., 2019; Hong et al., 2020). Ding et al. (2016) demonstrated that SSRP1 was down-regulated by miR-497, displaying an inhibited characteristics of proliferation and migration in HCC. It was also reported that miR-497 inhibited HCC metastasis by targeting NF-κB/SALL4 axis (Zhao et al., 2019). MiR-1246 is considered to act as an oncogene, which is up-regulated in the majority of cancers, stimulating tumor growth through affecting genes functioning in proliferation, apoptosis, and metastasis (Peng et al., 2019; Xu et al., 2019; Lin et al., 2020). It was described that the expression level of miR-1246 was significantly increased in HCC tissue, which was consistent with the *in vitro* test on HCC cell lines (Sun et al., 2014; Chuma et al., 2019; Huang et al., 2020).

Circulating miRNAs have been proven to be stable in peripheral blood so that it is feasible to extract circulating miRNAs from patients suffer cancer (Heneghan et al., 2010; Moshiri et al., 2018; Kovesdi et al., 2020). Further, the mini-traumatic advantage of blood samples makes a continuous test of circulating miRNAs *in vitro* possible while also guarantees good repeatability (Pratama et al., 2020). Therefore, abnormal expression levels of circulating miRNAs can serve as potential biomarkers for disease diagnosis, individualized treatment, and prognosis monitoring (Sharova et al., 2016; Todeschini et al., 2017; Valihrach et al., 2020).

Accordingly, through investigating serum expression levels of miR-497 and miR-1246 in HCC patients, our study aimed to explore the diagnosis value of miR-497 and miR-1246 in HCC and their relationship with the clinicopathological characteristics of HCC patients. In addition, this work also screened and enriched the target genes of miR-497 and miR-1246 to analyze the potential regulatory mechanisms of miR-497 and miR-1246 functions.

## Materials and Methods

### Clinical Samples

In total, 50 patients (30 males and 20 females) diagnosed with HCC from January 2014 to January 2015 in Huashan Hospital Affiliated to Fudan University were taken as the experimental group (HCC group). The ages ranged from 20 to 70, and the mean age was 45 ± 7.2 years. Based on TNM stages, the numbers of patients with TNM I + II and III were 24 and 26, respectively, and the numbers of patients with TNM N0 and N1 were 31 and 19, respectively. In terms of differentiation, there were 30 well and moderately differentiated cases and 20 poorly differentiated cases. The HCC group underwent follow-up surveys within 70 months after surgical resection. The control group consisted of 50 healthy subjects examined during the same period, including 28 males and 22 females. Sample sizes and power calculations were not performed for the study. The study design was approved by the Medical Ethics Committee of Huashan Hospital Affiliated to Fudan University and the informed consent forms were obtained in full.

### Standard Technique

The inclusion criteria were patients who were diagnosed with HCC based on pathological examinations and Nation Comprehensive Cancer Network (NCCN) clinical practice guidelines in hepatobiliary cancers.

The exclusion criteria were patients with other cancers or hematologic diseases; the occurrence of severe HCC complications or immune system diseases; confirmed poor compliance due to severe psychiatric disorders; and an unwillingness to participate in this research.

### Sample Preparation and Serum Total RNA Extraction

In total, 5 mL elbow vein blood was adopted from each patient, centrifuged at 4,000 rpm for 10 min to separate the serum into a clean EP tube, and stored at −80°C. Serum total RNA was separated and purified using the TRIzol method. Analyzing the quality and concentration of total RNA by NanoDrop 2000 spectrophotometer (Thermo Fisher Scientific, inc.). Agarose gel electrophoresis was applied to ensure the integrity of the total RNA. Storing total RNA at −80°C for RT-qPCR.

### RT-qPCR

In sum, 1 μg total RNA was reversely transcribed into cDNA using miScript II RT Kit (Qiagen GmbH) at 37°C for 1 h and then at 95°C for 5 min. Synthesized cDNA samples were stored at −80°C. The RT-qPCR was carried out strictly according to the miScript SYBR-Green PCR Kit (Qiagen GmbH) and each data was repeated three times. Caenorhabditis elegans miR-39 (cel-miR-39) was adopted as the control. The amplification reaction program was as follows: initial denaturation at 95°C for 15 min, then 40 cycles with each cycle at 94°C for 15 s followed by annealing at 55°C for 30 s, finally extended at 70°C for 30 s. The primers for RT-qPCR were as follows: miR-497 forward, “5′-CAGCAGCACACUGUGGUUUGU-3′”; miR-1246 forward, “5′-AAUGGAUUUUUGGAGCAGG-3′”; cel-miR-36 forward, “5′-UCACCGGUGUAAAUCAGCUUG-3′”; and miScript universal reverse primer, “5′-AGCCGAAGTGAGCCACTGAA-3′.” The 2−ΔΔCT (cycle threshold) method was used to obtain the relative expression levels of miRNAs.

### Serum AFP Quantification

Serum AFP level was quantified using an electron chemistry luminescence immunity analyzer (Roche Diagnostics) according to the manufacturer’s instruction. The reference value of serum AFP was 0–7 ng/mL.

### Bioinformatics Analysis

MiRDB and mirDIP databases were used to predict the potential target genes of miR-497 and miR-1246. The differential expression of potential target genes of miR-497 and miR-1246 in HCC was obtained from the GEPIA website^1^. The GO function, KEGG, and REACTOME pathways were used for enrichment analysis. The prognostic effects of potential target genes of miR-497 and miR-1246 in HCC were obtained from the Kaplan-Meier Plotter website^2^.

### Statistical Analysis

Statistical analysis was carried out using SPSS 19.0 (IBM Co.), and the results were presented as the mean ± standard error. Figures were depicted by GraphPad Prism 7.0 and Photoshop CS6 software. Statistical analysis between two groups was performed using Student’s *t*-test, and analysis between multiple groups was assessed by one-way analysis of variance (ANOVA) followed by a Student-Newman-Keuls *post hoc* analysis. Correlation analysis was performed using the Spearman rank test. Independent-Sample *t*-test was applied to analyze the values between two groups. ROC analysis was used to evaluate the diagnostic efficacy of miR-497, miR-1246, and AFP in distinguishing HCC patients from controls. The survival curve was displayed using the Kaplan-Meier method. *P* < 0.05 was considered significant.

## Results

### General Information of Subjects

The general information of subjects in the HCC group and control group is listed in Table 1. There is no significance between subjects from the two groups in terms of basic clinical data including gender, age, body mass index (BMI), drinking history, smoking history, and education level (*P* > 0.05).

**TABLE 1:** General information of the HCC group and the control group.

Characteristics	Control (*n* = 50)	HCC (*n* = 50)	*t*/*X*^2^	*P* value
**Sex**			0.1642	0.685
Male	28 (56.0%)	30 (60.0%)		
Female	22 (44.0%)	20 (40.0%)		
Age(year)	43.40 ± 11.93	42.30 ± 12.70	0.4463	0.6564
BMI(Kg/m^2^)	22.04 ± 2.80	21.68 ± 3.70	0.5487	0.5845
**Drinking history**			0.6568	0.418
Yes	31 (62.0%)	27 (54.0%)		
No	19 (38.0%)	23 (46.0%)		
**Smoking history**			0.3636	0.5460
Yes	29 (58.0%)	26 (52.0%)		
No	21 (42.0%)	24 (48.0%)		
**Education level**			0.6667	0.4140
>High school	28 (56.0%)	32 (64.0%)		
≤High school	22 (44.0%)	18 (36.0%)		

### Expression Levels of miR-497 and miR-1246 in Tissue and Serum of HCC Patients

**FIGURE 1:**
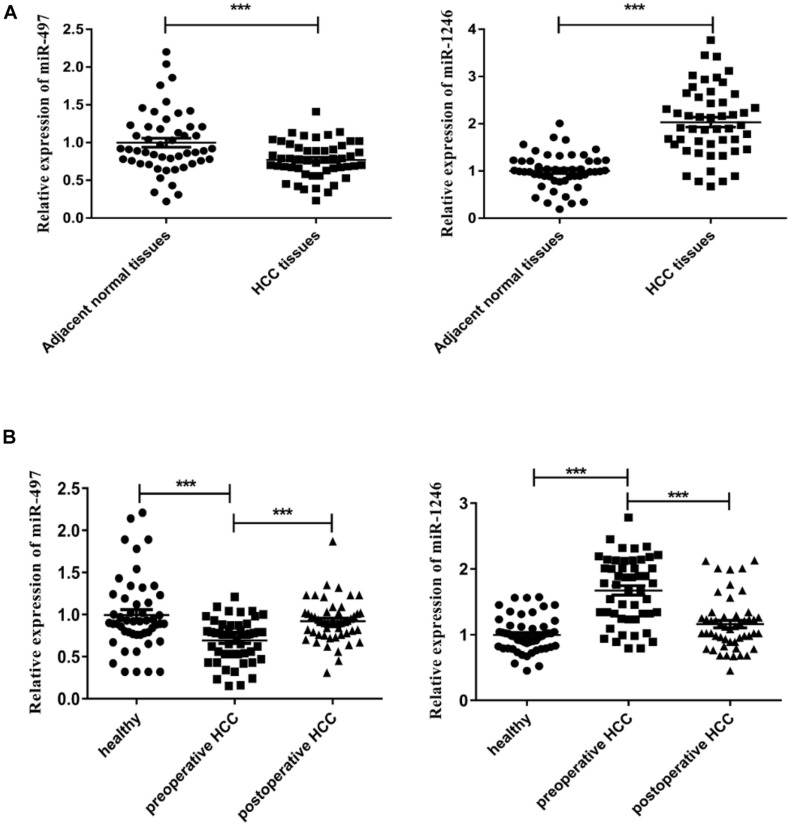
Expression levels of miR-497 and miR-1246 in HCC tissue and serum. **(A)** Relative expression levels of miR-497 (left) and miR-1246 (right) in HCC tissue and paired adjoint tissue. **(B)** Relative expression levels of miR-497 (left) and miR-1246 (right) in preoperative, postoperative, and healthy serum. ***P < 0.001.

In our study, the expression levels of miR-497 and miR-1246 in carcinoma tissue and adjoint tissue of each subject in the HCC group were tested. The results showed that the expression level of miR-497 in the HCC tissue was significantly down-regulated when compared with the paired adjoint tissue (*P* < 0.001, Figure 1A left) while the miR-1246 revealed a significantly higher expression level in carcinoma tissue (*P* < 0.001, Figure 1A right). The same expression levels profile was discovered in the serum. Compared to postoperative serum and the controls, preoperative serum miR-497 level was significantly down-regulated (*P* < 0.001, Figure 1B left) while miR-1246 significantly increased (*P* < 0.001, Figure 1B right). Thus, the results indicated the involvement of miR-497 and miR-1246 in the tumorigenesis and development of HCC as well as the potentiality of serving as biomarkers in early diagnosis of HCC.

### Correlation Between Levels of miR-497 and miR-1246 in HCC Pathologic Specimens and Clinicopathological Characteristics of HCC Patients

Further research focused on the statistical analysis between tissue levels of miR-497 and miR-1246 and clinicopathological characteristics of HCC patients, as shown in Tables 2, 3. The HCC tissue level of miR-497 was associated with tumor size (*P* = 0.047), TNM staging (*P* = 0.046), differentiation (*P* = 0.011), and metastasis (*P* = 0.002), but it was independent of gender, age, α-fetoprotein (AFP) level, condition of virus infection, alanine aminotransferase (ALT) level, aspartate aminotransferase (AST) level, and cirrhosis (*P* > 0.05). The MiR-1246 level was found to be correlated with TNM staging (*P* = 0.024), differentiation (*P* = 0.010), and metastasis (*P* = 0.011), and all factors showed statistical differences. Gender, age, AFP level, condition of virus infection, ALT level, AST level, cirrhosis, and tumor size were unrelated to miR-1246 level (*P* > 0.05).

**TABLE 2:** Correlation between expression levels of miR-497 in HCC tissue and clinicopathological characteristics of patients with hepatocellular carcinoma.

Clinicopathologic Parameters	Low Expression of miR-497 (*n* = 25)	High Expression of miR-497 (*n* = 25)	*P* value
**Gender**			
male	14	16	0.817
female	10	10	
**Age (year)**			
≤ 55	16	14	0.564
> 55	9	11	
**AFP(ng/ml)**			
≤ 7	12	17	0.152
> 7	13	8	
**Viral infection**			
with	16	10	0.089
without	9	15	
**ALT(U/L)**			
≤ 40 U/L	10	16	0.089
> 40 U/L	15	9	
**AST(U/L)**			
≤ 40 U/L	10	16	0.089
> 40 U/L	15	9	
**Cirrhosis**			
with	14	10	0.258
without	11	15	
**Tumor size(cm)**			
≤ 5	10	17	0.047^∗^
> 5	15	8	
**TNMstage**			
I-II	9	17	0.046^∗^
III-IV	16	8	
**Differentiation**			
middle or high	8	17	0.011^∗^
low	17	8	
**Metastasis**			
positive	20	9	0.002^∗∗^
negative	5	16	

*P < 0.05, **P < 0.01.

**TABLE 3:** Correlation between expression levels of miR-1246 in HCC tissue and clinicopathological factors of patients with hepatocellular carcinoma.

Clinicopathologic Parameters	Low Expression of miR-1246 (*n* = 25)	High Expression of miR-1246 (*n* = 25)	*P* value
**Gender**			
male	15	15	1
female	10	10	
**Age (year)**			
≤55	15	16	0.771
>55	10	9	
**AFP(ng/ml)**			
≤7	14	9	0.156
>7	11	16	
**Viral infection**			
with	12	17	0.152
without	13	8	
**ALT(U/L)**			
≤40 U/L	15	9	0.089
>40 U/L	10	16	
**AST(U/L)**			
≤40 U/L	14	10	0.258
>40 U/L	11	15	
**Cirrhosis**			
with	10	16	0.089
without	15	9	
**Tumor size(cm)**			
≤5	13	10	0.395
>5	12	15	
**TNM stage**			
I-II	17	9	0.024^∗^
III-IV	8	16	
**Differentiation**			
middle or high	15	6	0.010^∗∗^
low	10	19	
**Metastasis**			
positive	9	18	0.011^∗^
negative	16	7	

*P < 0.05; ***P < 0.01.

### Diagnostic Values of Applied Joint Detection of Serum miR-497, miR-1246, and AFP Levels to HCC Patients and Healthy Controls

**FIGURE 2:**
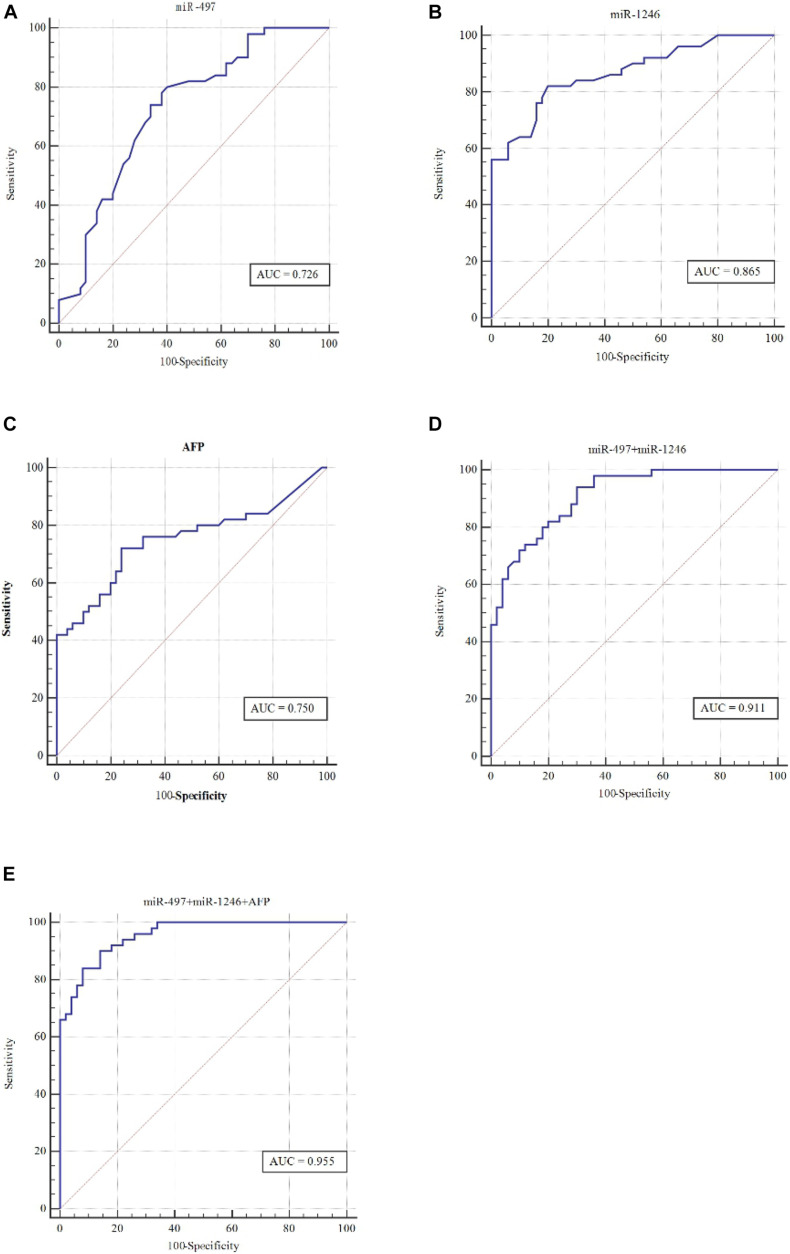
ROC curve can distinguish HCC patients from the control. Compared with 50 healthy controls, the ROC curve evaluated the diagnostic performances of serum **(A)** miR-497, **(B)** miR-1246, **(C)** AFP, **(D)** miR-497 + miR-1246, and **(E)** miR-497 + miR-1246 + AFP.

For determining the diagnostic values of serum miR-497, miR-1246, and AFP levels, ROC curves were drawn to analyze the Area Under Curve (AUC), sensitivity, and specificity (Figure 2 and Table 4). The AUC of serum miR-497 for HCC diagnosis was 0.726 [95% confidence interval (CI) is 0.628–0.810] with a sensitivity and specificity of 74.0 and 66.0%. The AUC of serum miR-1246 for HCC diagnosis was 0.865 (95% CI is 0.783 to 0.925) with a sensitivity and specificity of 82.0 and 80.0%. For further research, the ROC curve of the combination of miR-497 and miR-1246 for HCC diagnosis was drawn and the AUC was calculated to be 0.750 (95% CI is 0.653 to 0.831) with a sensitivity and specificity of 94.0 and 70.0%. Intriguingly, when AFP was taken together with the combination of miR-497 and miR-1246, the most robust capacity of discriminating HCC was shown whose AUC was 0.955 (95% CI is 0.837 to 0.958) with a sensitivity and specificity of 94 and 86.0%.

**TABLE 4:** AUC and the corresponding 95% CI of serum microRNA and AFP in patients with hepatocellular carcinoma compared with healthy controls.

Tumor markers	Sensitivity(%)	Specificity(%)	AUC	SE	95% CI	*P* value
miR-497	74.0	66.0	0.726	0.0511	0.628–0.810	< 0.001
miR-1246	82.0	80.0	0.865	0.0360	0.783–0.925	< 0.001
AFP	72.0	76.0	0.750	0.0507	0.653–0.831	< 0.001
miR-497 + miR-1246	94.0	70.0	0.911	0.0269	0.837–0.958	< 0.001
miR-497 + miR-1246 + AFP	90.0	86.0	0.955	0.0170	0.894–0.986	< 0.001

### Correlation Between Serum Levels of miR-497 and miR-1246 and Prognosis of HCC Patients

**FIGURE 3:**
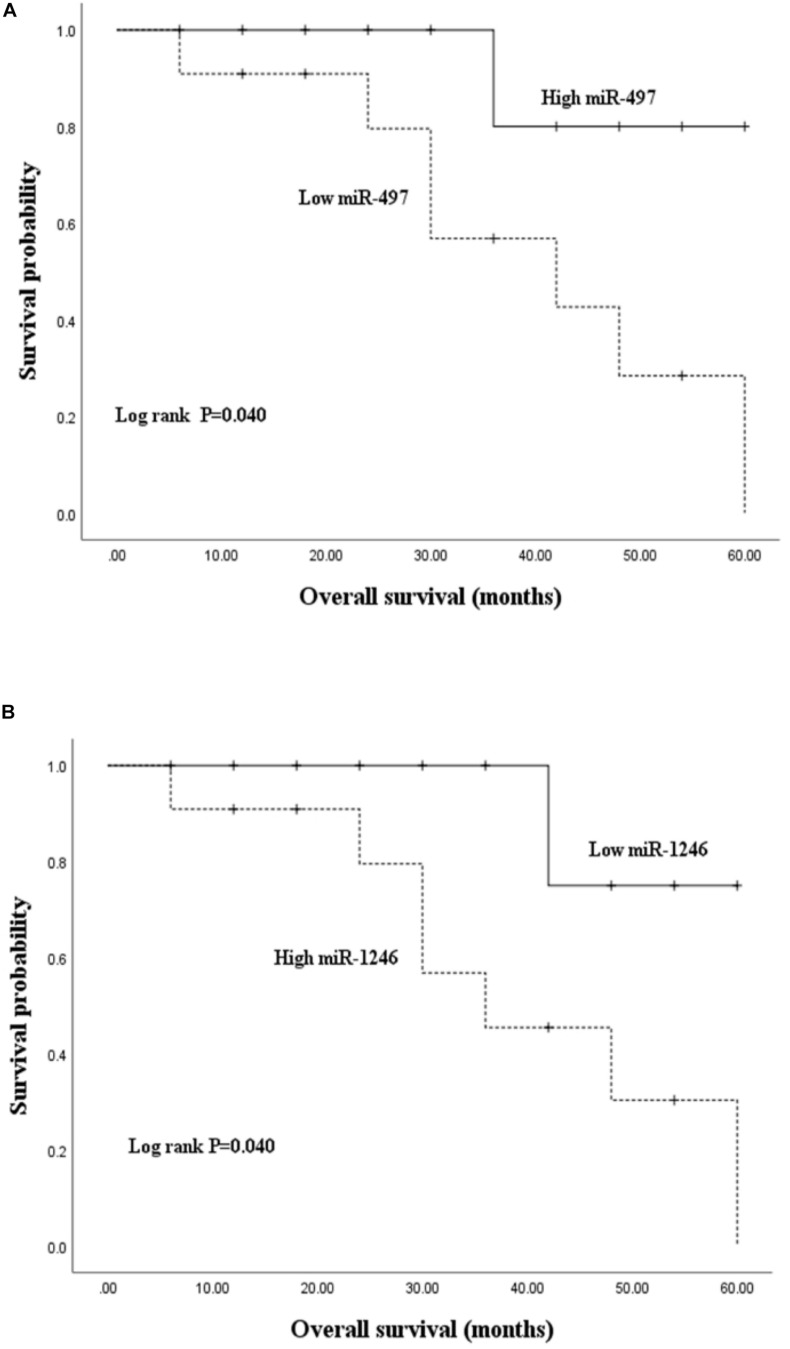
Kaplan-Meier overall survival curve was made based on preoperative serum miR-497 **(A)** and miR-1246 **(B)** relative expression levels. The high expression group and low expression group were founded on the median expression level.

To unveil the potential value of miR-497 and miR-1246 in prognosis monitoring, a survival analysis was conducted on HCC patients. Kaplan-Meier survival curves were depicted to assess the relationship between high or low miRNAs expression levels and the general survival situations of HCC patients. The median survival time of the HCC group was 46.527 months (Table 5). Kaplan-Meier analysis demonstrated that a low level of miR-497 was significantly related to the decreased survival probability of HCC patients (*P* = 0.040) (Figure 3A). However, the miR-1246 level showed a contrary tendency that a high level of miR-1246 was significantly related to the decreased survival probability (*P* = 0.040) (Figure 3B). The results indicated the potential values of miR-497 and miR-1246 in the prognostic evaluation of HCC patients.

**TABLE 5:** Correlation between serum miR-497 and miR-1246 levels and survival duration of HCC patients.

miRNA	miRNA expression level	survival time (month)	*P* value
	low expression	39.920	0.040^∗^
miR-497	high expression	55.200	
	total	46.527	
	low expression	55.50	0.040^∗^
miR-1246	high expression	39.636	
	total	46.527	

### Exploration of miR-497 and miR-1246 Target Gene

**FIGURE 4:**
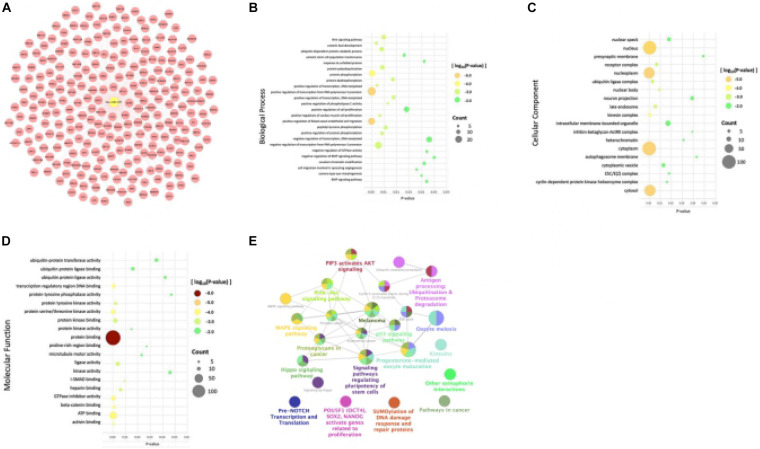
Databases, gene ontology terms, and the KEGG pathway were used to enrich the potential target genes of miR-497. **(A)** The predicted target genes of miR-497 were obtained from the database. **(B)** Target genes of miR-497 were significantly enriched in biological processes by BP analysis. **(C)** Target genes of miR-497 were significantly enriched in the essential components of cells by CC analysis. **(D)** The most significant result was protein binding, and at the same time, predicted genes were enriched in ATP binding, protein serine/threonine kinase activity for MF analysis. **(E)** The predicted target genes of miR-497 were mainly concentrated in some signal pathways.

**FIGURE 5:**
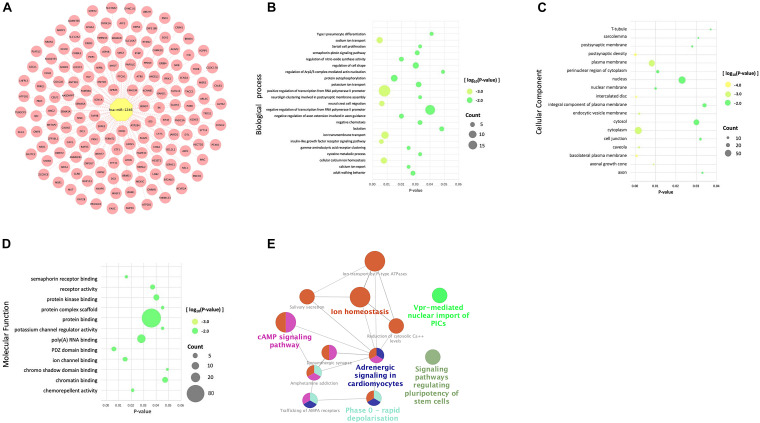
Databases, gene ontology terms, and the KEGG pathway were used to enrich the potential target genes of miR-1246. **(A)** The predicted target genes of miR-1246 were obtained from the database. **(B)** Target genes of miR-1246 were significantly enriched in biological processes by BP analysis. **(C)** Target genes of miR-1246 were significantly enriched in the essential components of cells by CC analysis. **(D)** The most significant result was protein binding, and at the same time, predicted genes were enriched in protein binding, and also expressed in poly (a) RNA binding, protein kinase binding, and chromatin binding. **(E)** The predicted target genes of miR-1246 were mainly concentrated in some signal pathways.

In order to investigate the mechanism of action of hsa-miR-497 and hsa-miR-1246, we utilized online databases to predict their target genes. We took the intersection of the results of miRDB and mirDIP databases and analyzed the target genes. Go function annotation included biological process (BP), cell composition (CC), molecular function (MF). And the KEGG and REACTOME pathways enrichment analysis were performed. To have a better understanding, all the results were visualized (Figures 4, 5).

A total of 258 predicted target genes of miR-497 were obtained from the database (Figure 4A). It should be noted that because the number of possible target genes of miR-497 is large, we chose 258 genes with high possibility (Target Score ≥ 90). BP analysis showed that the target genes of miR-497 were significantly enriched in biological processes like positive regulation of transcription from RNA polymerase II promoter and positive regulation of transcription, DNA template, etc. (Figure 4B). CC analysis showed that target genes were significantly enriched in the essential components of cells (Figure 4C). For MF analysis, the most significant result was protein binding, and, at the same time, predicted genes were enriched in ATP binding, protein serine/threonine kinase activity (Figure 4D), etc. The predicted target genes of miR-497 were mainly concentrated in PI3K/Akt signal pathway, proteoglycan in cancer and p53 signal pathway (Figure 4E), etc.

A total of 155 predicted target genes of miR-1246 were obtained from the database (Figure 5A). BP analysis showed that the target genes were mainly enriched in positive and negative regulation of transcription from RNA polymerase II promoter, ion transmembrane transport (Figure 5B). CC analysis showed that the predicted target genes of miR1246 were significantly expressed in the cytoplasm, plasma membrane, nucleus, nucleoplasm, cytosol, and other cell components (Figure 5C). MF analysis showed that miR-1246 was significantly enriched in protein binding, and it was also expressed in poly (a) RNA binding, protein kinase binding, and chromatin binding (Figure 5D). KEGG and REACTOME pathways analysis showed that the target genes were mainly enriched in the cAMP signaling pathway, adrenergic signaling in cardiomyocytes, ion homeostasis, and so on (Figure 5E).

### The Expression Levels and Prognostic Analysis of miR-497 and miR-1246 Target Genes

**FIGURE 6:**
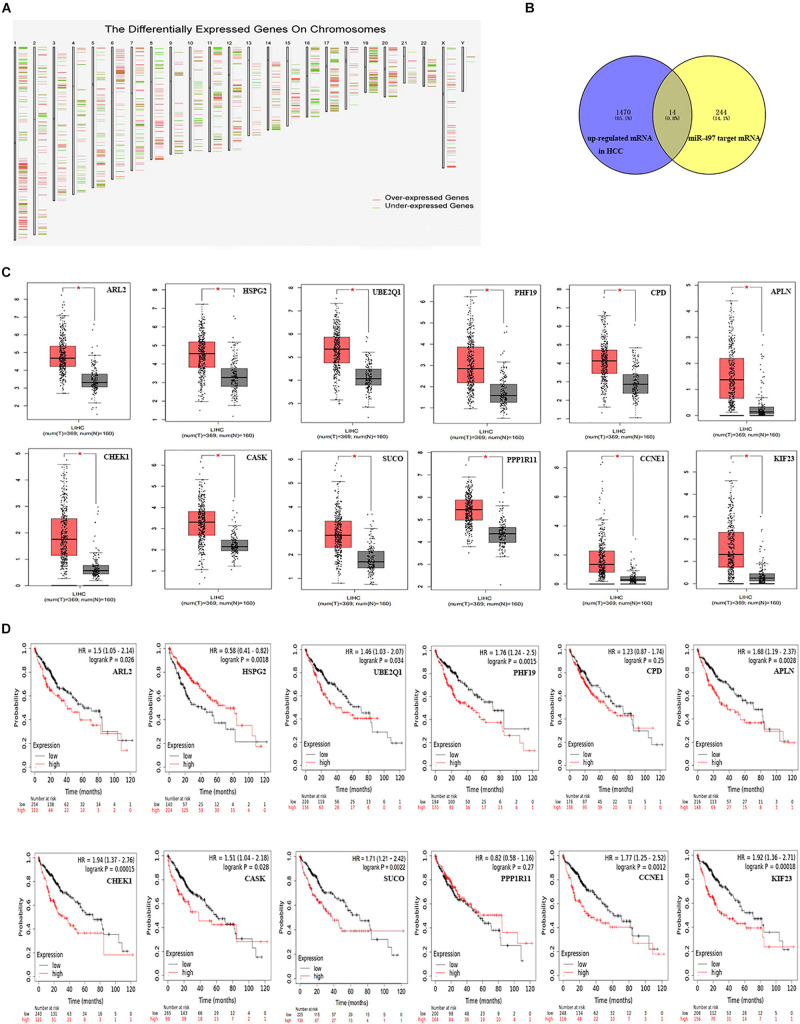
Identify the candidate targeted genes of miR-497 and its prognosis analysis. **(A)** Differentially expressed genes between HCC samples and normal samples from the GEPIA database. **(B)** The intersection of miR-497 target genes and the differentially expressed genes down-regulated in the GEPIA database were obtained. **(C)** The expression levels of ARL2, HSPG2, UBE2Q1, PHF19, CPD, APLN, CHEK1, CASK, SUCO, PPP1R11, CCNE1, KIF23, TMEM183A, and FASN between HCC samples and normal samples were obtained from the GEPIA database. **(D)** The prognostic analysis results of ARL2, HSPG2, UBE2Q1, PHF19, CPD, APLN, CHEK1, CASK, SUCO, PPP1R11, CCNE1, KIF23, TMEM183A, and FASN in HCC were obtained from the Kaplan-Meier Plotter website.

As we all know, a lot of evidence supports a negative correlation between miRNA expression and target genes. We first used the GEPIA database to identify differentially expressed genes between hepatocellular carcinoma samples and normal samples (screening conditions are | Log2FC| Cutoff is 1, q-value Cutoff is 0.05). Subsequently, 1,484 up-regulated mRNAs and 733 down-regulated mRNAs were identified (Figure 6A). After a comprehensive analysis of the up-regulated mRNA and miR-497 target genes, we further identified 14 miR-497 target genes with up-regulated mRNA in hepatocellular carcinoma samples (Figure 6B). These genes are ARL2 (ADP ribosylation factor like GTPase 2), HSPG2 (heparan sulfate proteoglycan 2), UBE2Q1 (ubiquitin-conjugating enzyme E2 Q1), PHF19 (PHD finger protein 19), CPD (carboxypeptidase D), APLN (apelin), CHEK1 (checkpoint kinase 1), CASK (calcium/calmodulin-dependent serine protein kinase), SUCO (SUN domain-containing ossification factor), PPP1R11 (protein phosphatase 1 regulatory inhibitor subunit 11), CCNE1 (cyclin E1), KIF23 (kinesin family member 23), TMEM183A (transmembrane protein 183A), and FASN (fatty acid synthase). As shown in Figure 6C, the expression levels of 14 genes in hepatocellular carcinoma samples were significantly higher than those in normal samples, and the difference was statistically significant (*P* < 0.05). The Kaplan-Meier Plotter website was used to evaluate the prognostic role of these 14 genes in hepatocellular carcinoma. As shown in Figure 6D, the low expression of ARL2, UBE2Q1, PHF19, APLN, CHEK1, CASK, SUCO, CCNE1, and KIF23 has a better prognosis and has statistical differences.

**FIGURE 7:**
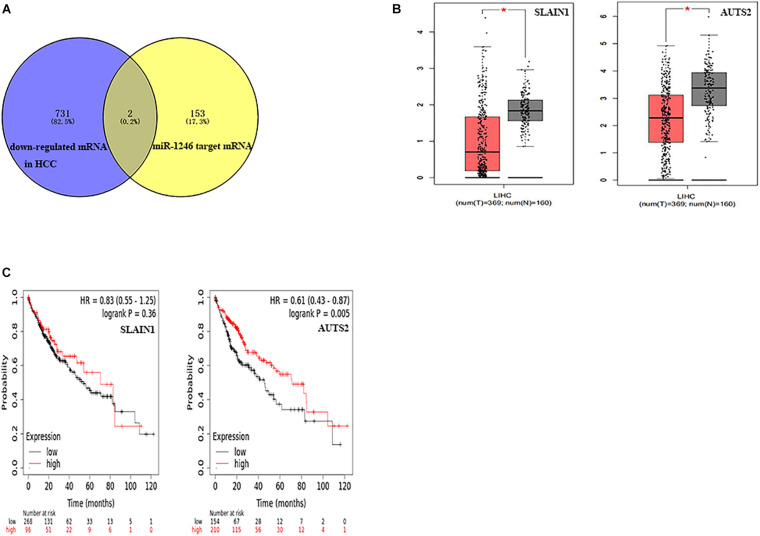
Identify the candidate target genes of miR-1246 and its prognosis analysis. **(A)** The intersection of miR-1246 target genes and the differentially expressed genes down-regulated in the GEPIA database were obtained. **(B)** The expression levels of AUTS2 and SLAIN1 between HCC samples and normal samples were obtained from the GEPIA database. **(C)** The prognostic analysis results of AUTS2 and SLAIN1 in HCC were obtained from the Kaplan-Meier Plotter website.

Similarly, after the comprehensive analysis of down-regulated mRNA and miR-1246 target genes, we further identified two miR-1246 target genes with down-regulated mRNA in hepatocellular carcinoma samples (Figure 7A). These genes are AUTS2 (activator of transcription and developmental regulator AUTS2) and SLAIN1 (SLAIN motif family member 1). As shown in Figure 7B, the expression levels of these two genes in hepatocellular carcinoma samples were significantly lower than normal tissues, and the difference was statistically significant (*P* < 0.05). The Kaplan-Meier Plotter website was used to evaluate the prognostic role of these two genes in hepatocellular carcinoma. As shown in Figure 7C, the high expression of AUTS2 has a better prognosis, and there is a statistical difference (*P* < 0.01).

Based on these findings, a potential miR-497-mRNA or miR-1246-mRNA regulatory network can be established, namely, miR-497-ARL2/UBE2Q1/PHF19/APLN/CHEK1/CASK/SUCO/CCNE1/KIF23, or miR-1246-AUTS2/SLAIN1, which contributes to the occurrence and development of hepatocellular carcinoma.

For requirements for a specific article type please refer to the Article Types on any Frontiers journal page. Please also refer to Author Guidelines for further information on how to organize your manuscript in the required sections or their equivalents for your field^3^.

## Discussion

Hepatocellular carcinoma constitutes more than 90% of liver cancer cases and is considered to be related to various etiological factors, which mainly include hepatitis B virus, hepatitis C virus, and alcohol abuse (Luu et al., 2020). HCC has an insidious onset and a slow progression stage, making the early clinical manifestations atypical. A large proportion of HCC patients are not properly diagnosed until the mid-late stage, which means the loss of the best treatment period (Yang and Heimbach, 2020). Thus, requirements for elevating early diagnostic rate and overall survival probability are being stressed by clinical doctors. For now, the main treatment methods of early-stage HCC are still surgical. In recent years, strategies of HCC remedy have been improved to some extent while the high postoperative complication rate remains unsolved (Mak et al., 2020). Therefore, discovering sensitive markers has great significance to early diagnosis and prognosis improvement of HCC.

Clinical diagnosis of HCC mainly depends on the comprehensive analysis of hepatitis virus infection condition, cirrhosis background, the non-specific clinical manifestation of the digestive system, laboratory indexes, and radiology data (Forner et al., 2018). However, due to the invasive procedures and the relative lack of sensitivity and specificity, it is urgently necessary for us to acquire non-invasive biomarkers to detect HCC. MiRNAs can stably exist in circulation by forming inclusion bodies and exosomes, which makes it possible to obtain and test miRNAs from biological fluid, serum, and stool samples (Valihrach et al., 2020). Thus, serum miRNAs have been considered promising biomarkers for HCC diagnosis.

Massive miRNAs have been proved to be associated with cancer progression and are differently expressed among normal tissue and tumor tissue (Goodall and Wickramasinghe, 2020). Interestingly, miRNAs can serve as both oncogenes and tumor suppressor genes based on parameters including miRNA types and tumor classifications, participating in a series of biological processes and pushing forward immense influences on tumorigenesis, development, and metastasis (Anastasiadou et al., 2018).

Previous studies have already proved that miR-497 level is down-regulated in several cancers. For example, Yang et al. (2016) demonstrated that down-regulated miR-497 was closely associated with a more significant chemoresistance of cervical cancer through the overexpression of TKT, finally affected the reactive oxygen species (ROS), cell proliferation, and viability. Down-regulated miR-497 participates in various pathways by indirectly stimulating the expression levels of target genes. As displayed in the study of Duan et al. (2020) a low level of miR-497 increased the HMGA2 level, resulting in the elevated growth and invasive capacity of HCC cells *in vivo* and *in vitro*. Meanwhile, an un-regulated miR-1246 level has been revealed in many malignancies. In the research of Yang et al., a high level of intracellular miR-1246 reduced the GSK-3β level, followed by the activation of the Wnt/β-catenin pathway, accounting for the initiation and enhancement of Epithelial-Mesenchymal Transition (EMT), which finally led to the metastasis of lung cancer cells (Wu and Yan, 2020). The same mechanism and pathway were proven to exist in liver cancer stem cells by Chai et al. (2016) who laid the foundation of HCC drug resistance and tumor recurrence.

Our study has revealed that the expression of miR-497 was significantly down-regulated in HCC tissue and the preoperative serum while a significantly higher level of miR-1246 was detected in HCC tissue and preoperative serum than that in the healthy controls. In the meantime, the down-regulated miR-497 and up-regulated miR-1246 levels were closely associated with the TNM staging, differentiation, and metastasis condition of HCC, suggested that miR-497 and miR-1246 might play important roles in HCC onset and development with miR-497 acting as an oncogene and miR-1246 acting as an anti-oncogene, respectively.

It has been reported that miR-497 and miR-1246 can serve as biomarkers in various tumors. For instance, research by Du et al. (2015) demonstrated that down-regulated miR-497 not only played a key role in the anti-apoptosis effect in bladder cancer but was also being closely related to the tumor burden of bladder cancer patients. Shi et al. (2020) have discovered the overexpression of serum miR-1246 packaged in exosomes and the association with TNM staging, showing the potentiality of serving as a diagnostic biomarker in gastric cancer. In our study, the sensitivity and specificity of serum miR-497 in HCC diagnosis were 74.0 and 66.0%, respectively, while the sensitivity and specificity of serum miR-1246 in HCC diagnosis were 82.0 and 80.0%, respectively. Providing that combining serum miR-497 and miR-1246 together for HCC diagnosis, the sensitivity and specificity reach 94.0 and 70.0%, respectively. The most robust diagnostic efficacy was presented by the triad of miR-497, miR-1246, and AFP, with the AUC, sensitivity, and specificity of 0.955, 94.0, and 86.0%. All the evidence suggests that miR-497 and miR-1246 can behave as biomarkers with excellent sensitivity and specificity that might be empowered for the early diagnosis of HCC, and the diagnostic efficacy can be even higher when AFP is incorporated into the combination.

We also found that serum miR-497 and miR-1246 levels were not only related to the preoperative tumor-bearing condition of HCC but were also related to the prognostic outcomes of HCC patients. The low level of serum miR-497 and high level of serum miR-1246 were associated with an unfavorable prognosis of postoperative HCC patients. Nevertheless, we believe that validating the mechanisms behind the influences of miR-497 and miR-1246 on various clinical factors in HCC will help to elucidate the correlations better.

MiRNA exerts its effects by inhibiting the expression of multiple target mRNAs. Based on the known target gene databases (miRDB and mirDIP), it is predicted that 258 mRNAs are the targeted regulatory molecules of miR-497, and 155 mRNAs are the target genes of miR-1246. The rich results of the GO analysis, KEGG, and REACTOME pathways showed that most of the potential target genes of miR-497 are significantly related to transcription, and PI3K Akt signal pathway, proteoglycan in cancer, and p53 signal pathway are the majority. Most potential target genes of miR-1246 are related to positive and negative regulation of transcription from RNA polymerase II promoter and ion transmembrane transport. The PIcAMP signaling pathway, adrenergic signaling in cardiomyocytes, and ion homeostasis predominate in signaling pathways.

One miRNA can target multiple mRNAs, and target mRNAs are usually tissue-specific. In order to test the predictive power of miR-497 and miR-1246 in HCC and verify their potential target genes, the expression levels of these mRNAs were further checked through the GEPIA database. Interestingly, it was confirmed that fourteen genes (ARL2, HSPG2, UBE2Q1, PHF19, CPD, APLN, CHEK1, CASK, SUCO, PPP1R11, CCNE1, KIF23, TMEM183A, and FASN) in miR-497 are highly expressed in hepatocellular carcinoma. And nine of the genes (ARL2, UBE2Q1, PHF19, APLN, CHEK1, CASK, SUCO, CCNE1, and KIF23) are related to the overall survival time of hepatocellular carcinoma patients. Simultaneously, we predict that two genes (AUTS2 and SLAIN1) regulated by miR-1246 are very lowly expressed in hepatocellular carcinoma, and the high expression of the AUTS2 gene among them is correlated with the longer overall survival time of hepatocellular carcinoma patients.

As a member of the ADP ribosylation factor (ARF) subfamily, ARL2 is highly conserved and ubiquitous in eukaryotes. Recent studies have shown that the expression of ARL2 in hepatocellular carcinoma is significantly increased, involved in the cell cycle regulation and DNA replication process of hepatocellular carcinoma, and may be used as a prognostic indicator (Hass et al., 2016). UBE2Q1 is a specific E2 ubiquitin-conjugating enzyme, which acts as an oncogene in hepatocellular carcinoma. Studies have shown that *in vitro* experiments, inhibiting the content of UBE2Q1 can significantly inhibit the proliferation of hepatocellular carcinoma cells, and lead to G1 arrest in HepG2 and BEL-7404 cells (Chang et al., 2015). PHF19 is a member of the polycomb group (PcG) that encodes a protein that functions by maintaining the repressive transcription state of many developmental regulatory genes. In addition, it has been proven that PHF19 plays an important role in the molecular etiology of HCC. Overexpression of PHF19 can promote the migration, invasion, and proliferation of HCC cells *in vitro* and participate in the regulation of the expression of cancer-related proteins (Xu et al., 2015). APLN is the endogenous ligand of the seven transmembrane G protein-coupled receptor (GPCR) APJ (APLNR). It is often upregulated in primary HCC and can be activated by phosphatidylinositol 3-kinase/protein kinase B (PI3K/Akt) pathway. APLN plays carcinogenic a role in the development of hepatocellular carcinoma and is a promising drug target for the treatment of hepatocellular carcinoma (Chen et al., 2019). CHEK1 is an evolutionarily conserved Ser/Thr kinase that mediates cell cycle arrest after DNA damage, and studies have reported that the oncogene CHEK1 is overexpressed in HCC and is associated with poor prognosis (Xie et al., 2014). The SUCO gene is located at the 1q24.3 site of humans and encodes the ossification factor of the SUN domain. Studies have shown that SUCO is up-regulated in HCC tissues and is involved in the cell cycle, cell metabolism, and cell proliferation. The high expression of SUCO is significantly related to the low overall survival rate of HCC patients and may be a potential diagnostic biomarker for HCC (Yue et al., 2019). CCNE1 is the regulatory subunit of cyclin-dependent kinase 2 (CDK2), believed to control the transition from static cells to the cell cycle. Sonntag et al. (2018) found that genetic inactivation of CCNE1 prevented the development of hepatocellular carcinoma in mice to a large extent, and was the main driving force for the initiation of HCC induced by DEN. KIF23 is a member of the Kinesin superfamily proteins family. It transports membrane organelles and protein complexes in a microtubule and ATP-dependent manner and participates in the proliferation and cycle of hepatocellular carcinoma cells. The high expression of KIF23 is significantly related to the shorter overall survival and disease-free survival of hepatocellular carcinoma patients and is an independent prognostic factor for the poor prognosis of hepatocellular carcinoma (Li et al., 2020). CASK and AUTS2 genes have not yet been reported on the mechanism of their involvement in hepatocellular carcinoma. These two genes are more likely to become the new target genes of miR-497 or miR-1246 in hepatocellular carcinoma cells.

## Conclusion

In conclusion, miR-497 and miR-1246 may be involved in the onset and development of HCC. Serum miR-497 and miR-1246 have good diagnostic sensitivity and specificity, and the addition of AFP will further increase the sensitivity. Furthermore, miR-497 may inhibit the biological process of HCC by regulating these nine genes ARL2, UBE2Q1, PHF19, APLN, CHEK1, CASK, SUCO, CCNE1, and KIF23. MiR-1246 may promote the development of HCC by inhibiting AUTS2. Therefore, miR-497 and miR-1246 may be new biomarkers robustly related to HCC diagnosis.

## Data Availability Statement

The original contributions presented in the study are included in the article/supplementary material, further inquiries can be directed to the corresponding author/s.

## Ethics Statement

The studies involving human participants were reviewed and approved by Ethics Review Board of Huashan Hospital affiliated to Fudan University. The patients/participants provided their written informed consent to participate in this study.

## Author Contributions

YoL and YuL: conceptualization, data curation, and funding acquisition. SC: methodology and formal analysis. ZF and SW: software. SC, ZF, and SW: writing—original draft preparation. SC, YoL, and YuL: writing—review and editing. All authors read and agreed to the published version of the manuscript.

## Conflict of Interest

The authors declare that the research was conducted in the absence of any commercial or financial relationships that could be construed as a potential conflict of interest. The handling editor and reviewer, QL, declared a shared affiliation, with no collaboration, with several of the authors, XY and CY, at the time of review.

## Footnotes

1 http://gepia.cancer-pku.cn/index.html

2 http://kmplot.com/

3 For Original Research articles, please note that the Material and Methods section can be placed in any of the following ways: before Results, before Discussion, or after Discussion.

## References

[B1] Anastasiadou E. Jacob L. S. Slack F. J. (2018). Non-coding RNA networks in cancer. *Nat. Rev. Cancer* 18 5–18. 29170536 10.1038/nrc.2017.99 PMC6337726

[B2] Borel F. Konstantinova P. Jansen P. L. (2012). Diagnostic and therapeutic potential of miRNA signatures in patients with hepatocellular carcinoma. *J. Hepatol.* 56 1371–1383. 10.1016/j.jhep.2011.11.026 22314424

[B3] Chae D. K. Park J. Cho M. Ban E. Jang M. Yoo Y. S. (2019). MiR-195 and miR-497 suppress tumorigenesis in lung cancer by inhibiting SMURF2-induced TGF-beta receptor I ubiquitination. *Mol. Oncol.* 13 2663–2678. 10.1002/1878-0261.12581 31581360 PMC6887584

[B4] Chai S. Ng K. Y. Tong M. Lau E. Y. Lee T. K. Chan K. W. (2016). Octamer 4/microRNA-1246 signaling axis drives Wnt/beta-catenin activation in liver cancer stem cells. *Hepatology* 64 2062–2076. 10.1002/hep.28821 27639189

[B5] Chang R. Wei L. Lu Y. Cui X. Lu C. Liu L. (2015). Upregulated expression of ubiquitin-conjugating enzyme E2Q1 (UBE2Q1) is associated with enhanced cell proliferation and poor prognosis in human hapatocellular carcinoma. *J. Mol. Histol.* 46 45–56. 10.1007/s10735-014-9596-x 25311764

[B6] Chen H. Wong C. C. Liu D. Go M. Y. Y. Wu B. Peng S. (2019). APLN promotes. *Theranostics* 9 5246–5260. 31410213 10.7150/thno.34713 PMC6691573

[B7] Chuma M. Toyoda H. Matsuzaki J. Saito Y. Kumada T. Tada T. (2019). Circulating microRNA-1246 as a possible biomarker for early tumor recurrence of hepatocellular carcinoma. *Hepatol. Res.* 49 810–822. 30920086 10.1111/hepr.13338

[B8] Ding Q. He K. Luo T. Deng Y. Wang H. Liu H. (2016). SSRP1 contributes to the malignancy of hepatocellular carcinoma and is negatively regulated by miR-497. *Mol. Ther.* 24 903–914. 10.1038/mt.2016.9 26755331 PMC4881782

[B9] Dong G. Zhang S. Shen S. Sun L. Wang X. Wang H. (2020). SPATS2, negatively regulated by miR-145-5p, promotes hepatocellular carcinoma progression through regulating cell cycle. *Cell Death Dis.* 11 837. 10.1038/s41419-020-03039-y PMC7547105 33037180

[B10] Du M. Shi D. Yuan L. Li P. Chu H. Qin C. (2015). Circulating miR-497 and miR-663b in plasma are potential novel biomarkers for bladder cancer. *Sci. Rep.* 5:10437. 10.1038/srep10437 PMC4444850 26014226

[B11] Duan Y. Zhao M. Jiang M. Li Z. Ni C. (2020). LINC02476 Promotes the malignant phenotype of hepatocellular carcinoma by sponging miR-497 and increasing HMGA2 expression. *Onco Targets Ther.* 13 2701–2710. 10.2147/ott.s237069 32280244 PMC7132004

[B12] Forner A. Reig M. Bruix J. (2018). Hepatocellular carcinoma. *Lancet* 391 1301–1314. 29307467 10.1016/S0140-6736(18)30010-2

[B13] Gebert L. F. R. MacRae I. J. (2019). Regulation of microRNA function in animals. *Nat. Rev. Mol. Cell Biol.* 20 21–37. 10.1038/s41580-018-0045-7 30108335 PMC6546304

[B14] Goodall G. J. Wickramasinghe V. O. (2020). RNA in cancer. *Nat. Rev. Cancer* 21 22–36. 33082563 10.1038/s41568-020-00306-0

[B15] Hass H. G. Vogel U. Scheurlen M. Jobst J. (2016). Gene-expression analysis identifies specific patterns of dysregulated molecular pathways and genetic subgroups of human hepatocellular carcinoma. *Anticancer Res.* 36 5087–5095. 10.21873/anticanres.11078 27798868

[B16] Heneghan H. M. Miller N. Kerin M. J. (2010). Circulating miRNA signatures: promising prognostic tools for cancer. *J. Clin. Oncol.* 28 e575–e576. 10.1200/JCO.2010.29.8901 20697097

[B17] Hong S. Yan Z. Song Y. Bi M. Li S. (2020). LncRNA AGAP2-AS1 augments cell viability and mobility, and confers gemcitabine resistance by inhibiting miR-497 in colorectal cancer. *Aging* 12 5183–5194. 10.18632/aging.102940 32202509 PMC7138564

[B18] Huang J. L. Fu Y. P. Gan W. Liu G. Zhou P. Y. Zhou C. (2020). Hepatic stellate cells promote the progression of hepatocellular carcinoma through microRNA-1246-RORalpha-Wnt/beta-Catenin axis. *Cancer Lett.* 476 140–151. 10.1016/j.canlet.2020.02.012 32061951

[B19] Kovesdi A. Kurucz P. A. Nyiro G. Darvasi O. Patocs A. Butz H. (2020). Circulating miRNA increases the diagnostic accuracy of chromogranin A in metastatic pancreatic neuroendocrine tumors. *Cancers (Basel)* 12:2488. 10.3390/cancers12092488 32887459 PMC7565801

[B20] Li X. Huang W. Huang W. Wei T. Zhu W. Chen G. (2020). Kinesin family members KIF2C/4A/10/11/14/18B/20A/23 predict poor prognosis and promote cell proliferation in hepatocellular carcinoma. *Am. J. Transl. Res.* 12 1614–1639. 32509165 PMC7270015

[B21] Lin D. Xu H. P. Lin J. H. Hu H. H. Wang Q. Zhang J. (2020). Long non-coding RNA MIAT promotes non-small cell lung cancer progression by sponging miR-1246. *Eur. Rev. Med. Pharmacol. Sci.* 24:8626. 10.26355/eurrev_202009_22762 32965021

[B22] Luu H. N. Neelakantan N. Geng T. T. Wang R. Bee G. G. B. Clemente J. C. (2020). Quality diet indexes and risk of hepatocellular carcinoma: findings from the singapore chinese health study. *Int. J. Cancer* 148 2102–2114. 10.1002/ijc.33367 33129230 PMC11572543

[B23] Mak L. Y. Wong D. K. Pollicino T. Raimondo G. Hollinger F. B. Yuen M. F. (2020). Occult hepatitis B infection and hepatocellular carcinoma: epidemiology, virology, hepatocarcinogenesis and clinical significance. *J. Hepatol.* 73 952–964. 10.1016/j.jhep.2020.05.042 32504662

[B24] Moshiri F. Salvi A. Gramantieri L. Sangiovanni A. Guerriero P. De Petro G. (2018). Circulating miR-106b-3p, miR-101-3p and miR-1246 as diagnostic biomarkers of hepatocellular carcinoma. *Oncotarget* 9 15350–15364. 10.18632/oncotarget.24601 29632649 PMC5880609

[B25] Peng W. Li J. Chen R. Gu Q. Yang P. Qian W. (2019). Upregulated METTL3 promotes metastasis of colorectal cancer via miR-1246/SPRED2/MAPK signaling pathway. *J. Exp. Clin. Cancer Res.* 38:393. 10.1186/s13046-019-1408-4 PMC6729001 31492150

[B26] Pratama M. Y. Visintin A. Crocè L. S. Tiribelli C. Pascut D. (2020). Circulatory miRNA as a biomarker for therapy response and disease-free survival in hepatocellular carcinoma. *Cancers* 12:2810. 10.3390/cancers12102810 33003646 PMC7601056

[B27] Sharova E. Grassi A. Marcer A. Ruggero K. Pinto F. Bassi P. (2016). A circulating miRNA assay as a first-line test for prostate cancer screening. *Br. J. Cancer* 114 1362–1366. 10.1038/bjc.2016.151 27228285 PMC4984473

[B28] Shi Y. Wang Z. Zhu X. Chen L. Ma Y. Wang J. (2020). Exosomal miR-1246 in serum as a potential biomarker for early diagnosis of gastric cancer. *Int. J. Clin. Oncol.* 25 89–99. 10.1007/s10147-019-01532-9 31506750

[B29] Sonntag R. Giebeler N. Nevzorova Y. A. Bangen J. M. Fahrenkamp D. Lambertz D. (2018). Cyclin E1 and cyclin-dependent kinase 2 are critical for initiation, but not for progression of hepatocellular carcinoma. *Proc. Natl. Acad. Sci. U.S.A.* 115 9282–9287. 10.1073/pnas.1807155115 30150405 PMC6140539

[B30] Sun Z. Meng C. Wang S. Zhou N. Guan M. Bai C. (2014). MicroRNA-1246 enhances migration and invasion through CADM1 in hepatocellular carcinoma. *BMC Cancer* 14:616. 10.1186/1471-2407-14-616 25159494 PMC4150976

[B31] Todeschini P. Salviato E. Paracchini L. Ferracin M. Petrillo M. Zanotti L. (2017). Circulating miRNA landscape identifies miR-1246 as promising diagnostic biomarker in high-grade serous ovarian carcinoma: a validation across two independent cohorts. *Cancer Lett.* 388 320–327. 10.1016/j.canlet.2016.12.017 28017893

[B32] Treiber T. Treiber N. Meister G. (2019). Regulation of microRNA biogenesis and its crosstalk with other cellular pathways. *Nat. Rev. Mol. Cell Biol.* 20 5–20. 10.1038/s41580-018-0059-1 30728477

[B33] Valihrach L. Androvic P. Kubista M. (2020). Circulating miRNA analysis for cancer diagnostics and therapy. *Mol. Aspects Med.* 72:100825. 10.1016/j.mam.2019.10.002 31635843

[B34] Villanueva A. (2019). Hepatocellular carcinoma. *N. Engl. J. Med.* 380 1450–1462. 30970190 10.1056/NEJMra1713263

[B35] Wu H. Yan H. (2020). Expression and diagnostic value of miR-34c and miR-141 in serum of patients with colon cancer. *Oncol. Lett.* 20:98. 10.3892/ol.2020.11959 PMC7439149 32831917

[B36] Xie Y. Wei R. R. Huang G. L. Zhang M. Y. Yuan Y. F. Wang H. Y. (2014). Checkpoint kinase 1 is negatively regulated by miR-497 in hepatocellular carcinoma. *Med. Oncol. (Northwood Lond. Engl.)* 31:844. 10.1007/s12032-014-0844-4 24464213

[B37] Xu H. Hu Y. W. Zhao J. Y. Hu X. M. Li S. F. Wang Y. C. (2015). MicroRNA-195-5p acts as an anti-oncogene by targeting PHF19 in hepatocellular carcinoma. *Oncol. Rep.* 34 175–182. 10.3892/or.2015.3957 25955388

[B38] Xu Y. F. Hannafon B. N. Khatri U. Gin A. Ding W. Q. (2019). The origin of exosomal miR-1246 in human cancer cells. *RNA Biol.* 16 770–784. 10.1080/15476286.2019.1585738 30806147 PMC6546371

[B39] Yang H. Wu X. L. Wu K. H. Zhang R. Ju L. L. Ji Y. (2016). MicroRNA-497 regulates cisplatin chemosensitivity of cervical cancer by targeting transketolase. *Am. J. Cancer Res.* 6 2690–2699. 27904781 PMC5126283

[B40] Yang J. D. Heimbach J. K. (2020). New advances in the diagnosis and management of hepatocellular carcinoma. *BMJ (Clin. Res.)* 371:m3544. 10.1136/bmj.m3544 33106289

[B41] Yue C. Liang C. Ge H. Yan L. Xu Y. Li G. (2019). SUCO as a promising. *Med. Sci. Monit.* 25 6292–6303. 10.12659/msm.915262 31434866 PMC6716297

[B42] Zhang Z. Tan X. Luo J. Yao H. Si Z. Tong J. S. (2020). The miR-30a-5p/CLCF1 axis regulates sorafenib resistance and aerobic glycolysis in hepatocellular carcinoma. *Cell Death Dis.* 11:902. 10.1038/s41419-020-03123-3 PMC7584607 33097691

[B43] Zhao B. Wang Y. Tan X. Ke K. Zheng X. Wang F. (2019). Inflammatory micro-environment contributes to stemness properties and metastatic potential of HCC via the NF-kappaB/miR-497/SALL4 Axis. *Mol. Ther. Oncol.* 15 79–90. 10.1016/j.omto.2019.08.009 31650028 PMC6804787

